# High versus low dose tranexamic acid as part of a patient blood management strategy for reducing blood loss in patients undergoing surgery for adolescent idiopathic scoliosis

**DOI:** 10.1007/s43390-021-00387-3

**Published:** 2021-07-16

**Authors:** Sundeep Tumber, Adam Bacon, Casey Stondell, Sampaguita Tafoya, Sandra L. Taylor, Yashar Javidan, Eric Klineberg, Rolando Roberto

**Affiliations:** 1 Shriners Hospitals for Children, Northern California, Sacramento, CA, USA; 2 University of California, Davis, Sacramento, CA, USA; 3 Department of Public Health Sciences, School of Medicine, University of California, Davis, Sacramento, CA, USA

**Keywords:** Tranexamic Acid, Adolescent Idiopathic Scoliosis, Scoliosis, Patient Blood Management, Blood Loss

## Abstract

**Purpose:**

The administration of tranexamic acid (TXA) has been shown to be beneficial in reducing blood loss during surgery for adolescent idiopathic scoliosis (AIS), but optimal dosing has yet to be defined. This retrospective study compared high versus low dose TXA as part of a Patient Blood Management strategy for reducing blood loss in patients undergoing posterior spine fusion surgery.

**Methods:**

Clinical records were reviewed for 223 patients with AIS who underwent posterior spinal fusion of five or more levels during a six-year time period. We compared normalized blood loss, total estimated blood loss (EBL), and the need for transfusion between patients receiving high dose TXA (loading dose of ≥30 mg/kg) versus low dose TXA (loading dose <30 mg/kg). Both groups received maintenance TXA infusions of 10 mg/kg/hr until skin closure.

**Results:**

Patient demographics, curves, and surgical characteristics were similar in both groups. The high dose TXA group had a 36% reduction in normalized blood loss (1.8 cc/kg/level fused versus 2.8 cc/kg/level fused, p<0.001) and a 37.5% reduction in total EBL (1000 cc versus 1600 cc, p<0.001). Patients in the high dose group had a 48% reduction in PRBC transfusion, with only 19% receiving a transfusion of PRBC compared to 67% in the low dose group (p<0.001).

**Conclusion:**

When combined with other proven Patient Blood Management strategies, the use of high dose TXA compared to low dose TXA may be beneficial in reducing blood loss for patients with adolescent idiopathic scoliosis undergoing posterior spinal fusion surgery.

**Level of Evidence:**

Level III, retrospective cohort

## Introduction

Bleeding during surgery for adolescent idiopathic scoliosis (AIS) can be substantial with average blood loss reported to be between 750–1,500 cc, and up to 9.7% of patients requiring massive transfusion (transfusion of >100% total blood volume within 24 hours) [1–2]. Furthermore, data from the Pediatric Perioperative Cardiac Arrest Registry showed that spine surgery is one of the most at-risk surgery types for perioperative cardiac arrest, accounting for 41% of all arrests. Blood loss-induced hypovolemia and hyperkalemia secondary to transfusion were the most common contributing factors [3].

Tranexamic acid (TXA) is an antifibrinolytic agent which competitively inhibits the conversion of plasminogen to plasmin thereby preventing dissolution (fibrinolysis) of formed clot [4]. Even when Patient Blood Management (PBM) strategies are used, the addition of TXA has been shown to decrease blood loss by 27% and reduce transfusion requirements as compared to placebo for AIS surgery [5].

The optimal dosing regimen of TXA in surgery for AIS has yet to be defined. A recent meta-analysis of 334 patients with AIS showed that a high dose TXA regimen (loading dose of 50–100 mg/kg, maintenance infusion of 10–30mg/kg/hr) significantly reduced blood loss when compared to placebo and did not lead to any significant thromboembolic events [6].

The objective of our study is to assess the effects of different dosing regimens of TXA (high vs. low), as part of a Patient Blood Management strategy, on blood loss and transfusion requirements in patients undergoing posterior spinal fusion(PSF) surgery for AIS.

## Methods

The Western Institutional Review Board granted approval for this retrospective review (NCA 2015R, 9/21/2020). All patients who underwent PSF surgery of more than 5 vertebral levels between January 2014 and August 2020 at our institution were included in the electronic medical record review. A total of 640 patients were identified. We excluded patients undergoing anterior/posterior spinal fusion and patients with neuromuscular scoliosis. 239 patients met initial inclusion criteria.

Patients were then categorized as receiving either high or low dose TXA. The high dose group received a loading dose of ≥30 mg/kg, whereas the low dose group received a loading dose <30 mg/kg. The maximum loading dose was 5 grams. Both groups received maintenance TXA infusions of 10 mg/kg/hr until skin closure. 10 patients who did not receive a loading dose were excluded. Additionally, 6 patients who received loading doses of <10 mg/kg were excluded. The records of 223 patients met inclusion criteria.

### Outcomes

To account for patient weight and number of levels fused, we used normalized blood loss (NBL) as our primary outcome [7]. NBL is defined as (estimated blood loss/number of levels fused)/weight in kg. Secondary outcomes included total estimated blood loss (EBL), percent of blood volume lost, EBL per level fused, EBL per screw placed, and the need for intraoperative transfusion of packed red blood cells (PRBC). Blood volume was estimated by weight (kg) x 70 cc/kg.

The following patient demographic data were recorded: age, height, weight, and body mass index (BMI). Spine curvature characteristics, including the largest preoperative curve, largest bending curve, and percent curve flexibility were also recorded as they might reflect the difficulty of the surgical operation. Surgical characteristics included the number of Ponte osteotomies (radical facet joint resection) performed, number of screws placed, number of vertebral levels fused, postoperative major curve, percent curve correction, implant density, the use of halo-femoral traction, and operative time. Anesthetic characteristics included the loading dose of TXA (mg/kg) as well as the volume of crystalloid (cc) and albumin (grams) administered. The mean arterial pressure (MAP) was analyzed every 5 minutes from incision to pedicle screw electromyographic testing. Patient Blood Management strategies included the following:

1. The use of cell salvage for all cases.
2. TXA use, either high dose or low dose. In mid-2017, our protocol changed from a low dose TXA dosing strategy (loading dose <30 mg/kg) to a high dose TXA dosing strategy (loading dose of ≥30 mg/kg). Both groups received maintenance TXA infusions of 10 mg/kg/hr until skin closure.
3. Controlled hypotension: We used a target mean arterial pressure (MAP) of 60–65 mmHg from incision to pedicle screw electromyographic testing. The blood pressure was increased before rod placement and curve correction to preoperative baseline levels or higher.
4. Use of topical hemostatic agents (Gelfoam, bonewax, Surgifoam, Surgicel). One or more of these agents were used in all cases.
5. Use of ultrasonic bone scalpel (Misonix BoneScalpel) or bipolar sealer (Aquamantys) was used at the surgeon’s discretion.
6. A Jackson table was used for prone positioning.

The patients did not donate autologous blood and optimization of hemoglobin before surgery was not performed. Anesthesiologists transfused the patients at their discretion.

### Statistical Analysis

Quantitative traits were summarized as medians [25^th^ quantile, 75^th^ quantile] because many were markedly right skewed. Categorical traits were summarized as proportions. Wilcoxon rank-sum tests were used to compare quantitative traits between low and high dosing strategies. Chi-square tests or Fisher’s exact test were used to test for associations between dosing levels and categorical traits. All statistical analyses were conducted using R Statistical Software version 4.0.3 and evaluated at a two-sided significance level of 0.05.

## Results

### Quantitative Traits

Table 1 provides summary statistics and results of Wilcoxon rank-sum test comparing quantitative traits by dosing level. Patient demographics, curve characteristics, and surgical characteristics were similar in both groups except for operative time, which was longer in the low dose TXA group (357 minutes vs. 299 minutes, p<0.001). Anesthetic characteristics were also similar in both groups except for the grams of albumin given, with patients in the low dose group receiving more (37.5 grams vs. 25 grams, p<0.001). There were no thrombotic events or seizures in either group.

**Fig. 1:**
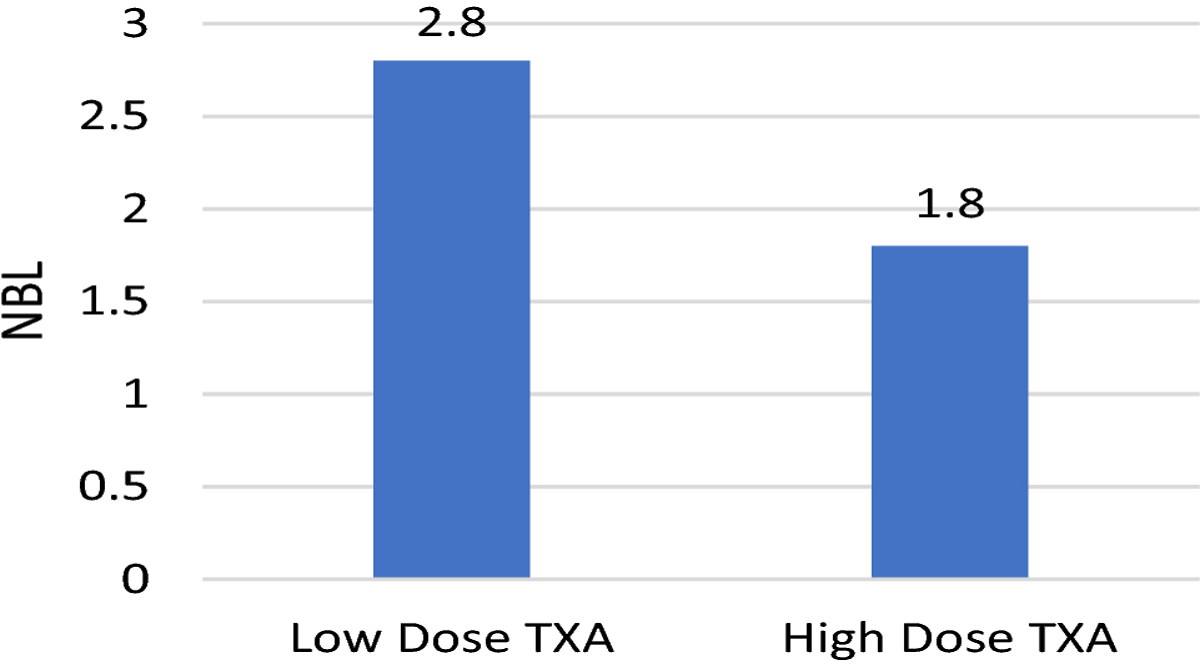
Effect of TXA Dosing on Normalized Blood Loss ((EBL/Levels Fused)/Kg)

**Fig. 2:**
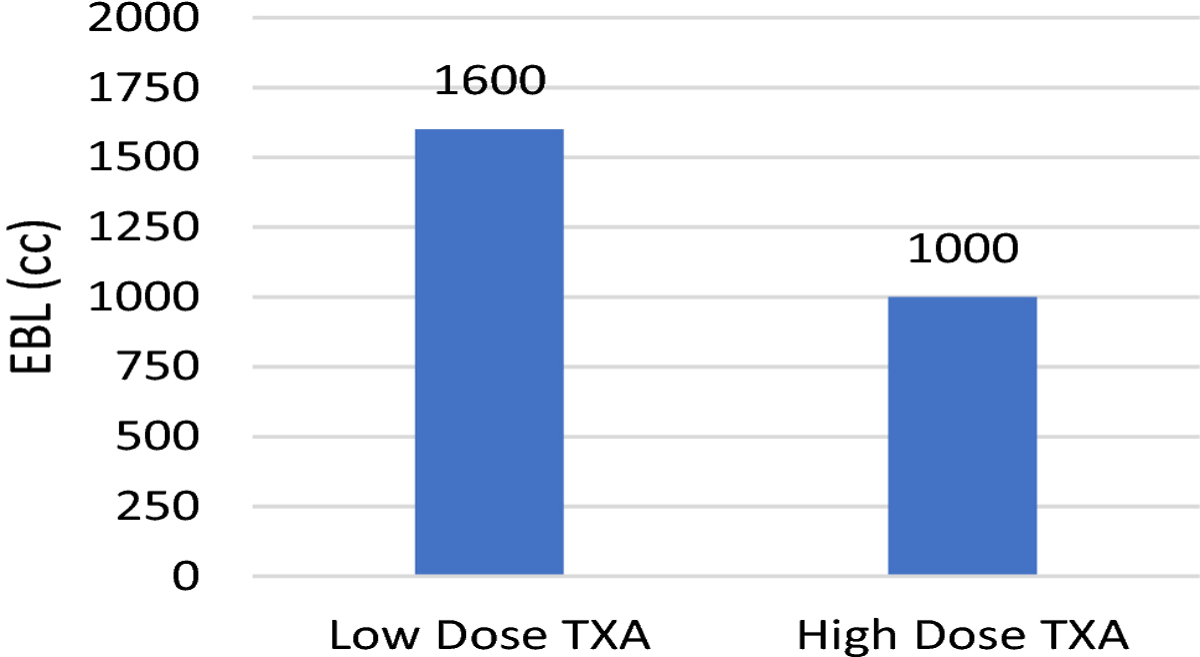
Effect of TXA Dosing on Estimated Blood Loss

**Fig. 3:**
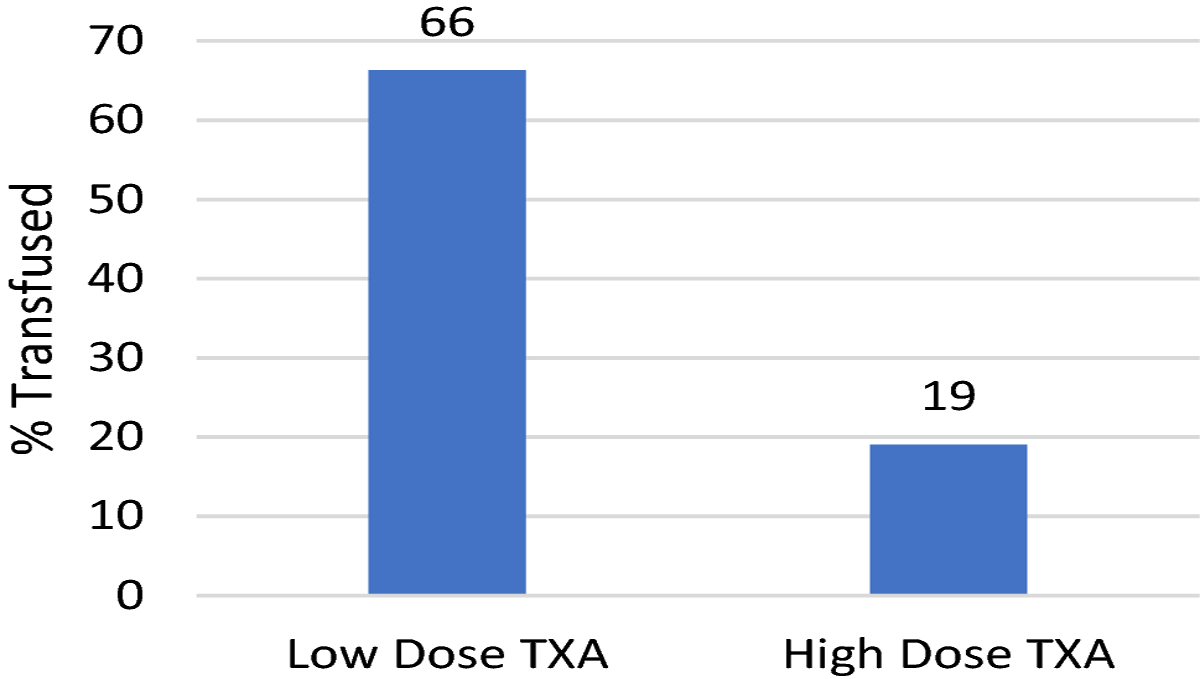
Effect of TXA Dosing on % of Patients Requiring PRBC Transfusion

Patients in the high dose TXA group had a 36% reduction in normalized blood loss from 2.8 cc/kg/level fused in the low dose TXA group to 1.8 cc/kg/level fused in the high dose TXA group (p<0.001). EBL decreased from 1600 cc in the low dose TXA group to 1000 cc in the high dose TXA group resulting in a 37.5% reduction (p<0.001). Patients in the high dose group had a 48% reduction in PRBC transfusion, with only 19% receiving a transfusion of PRBC compared to 67% in the low dose group (p<0.001). The results are summarized in Figures 1–3.

### Categorical Traits

Table 2 provides summary statistics and results of tests of association for categorical traits and dosing level. Both groups had a similar proportion of female patients and percent of patients that had Ponte osteotomies. The need for a blood transfusion was significantly reduced in the high dose TXA group (19%) vs the low dose TXA group (67%). The use of the bipolar sealer was not significant between groups. However, halo-femoral traction and the use of a BoneScalpel were higher in the high dose TXA group.

## Discussion

In our retrospective review of 239 patients with AIS undergoing PSF, blood loss and transfusion requirements were lower with use of the use of high dose TXA as part of a Patient Blood Management program. The high dose TXA group had a reduction in normalized blood loss of 36% and a reduced rate of PRBC transfusion from 67% to 19%. There were no reports of thrombotic events.

The optimal dosing regimen of TXA for controlling blood loss for scoliosis surgery for patients with AIS has yet to be determined. Our results indicate a benefit of a high dose TXA regimen are similar to a retrospective review by Johnson et al. of 166 patients with AIS undergoing PSF. Their study demonstrated that a high dose TXA regimen (50 mg/kg loading dose, maintenance dose of 5 mg/kg/hr) had significantly less intraoperative blood loss (by 30%) and a decreased RBC transfusion requirement (by 60%) when compared to a low dose regimen (10 mg/kg loading dose, maintenance dose of 1 mg/kg/hr) [8].

**Fig. 4:**
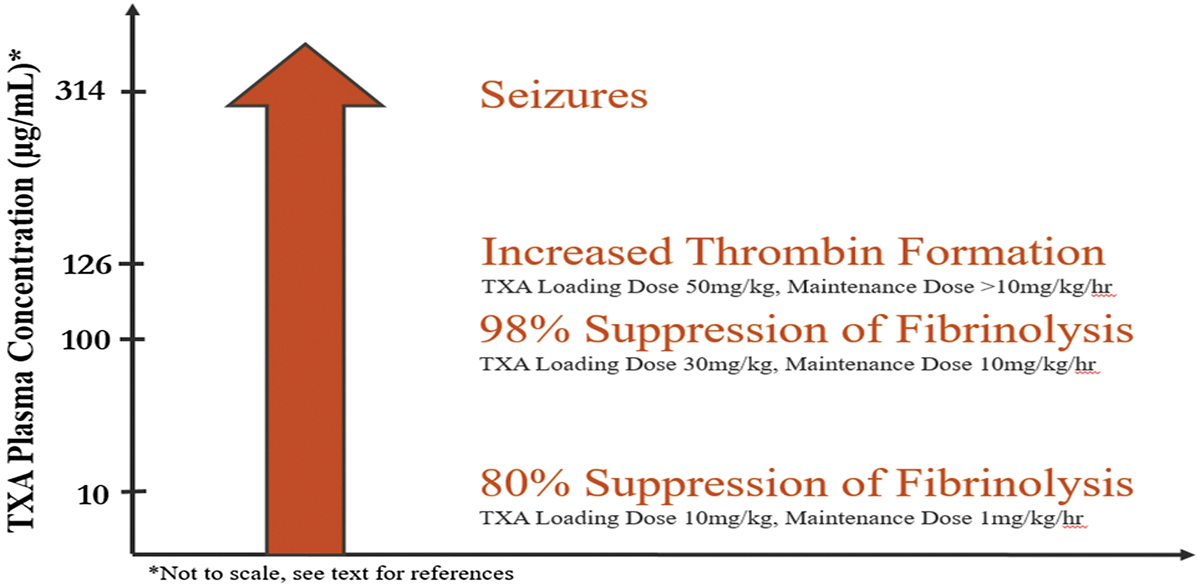
The effect of increasing TXA plasma concentration on hemostasis

The potential benefit of high dose TXA may be explained by the drug’s pharmacokinetic profile and the therapeutic plasma concentrations of different dosing regimens [9]. It is well known that TXA reduces bleeding by inhibiting fibrinolysis, but at higher doses, lesser-known mechanisms may come into play. A concentration-dependent reduction in bleeding by TXA’s additional roles in improving platelet function, aiding in clot stabilization, and increasing thrombin formation might explain the benefit of higher dosing [10–15]. TXA’s ability to suppress fibrinolysis by 80% can be obtained with a low plasma concentration (10 μg/ml), which can be reached with the administration of a 10 mg/kg loading dose, followed by a maintenance infusion of 1 mg/kg/hr [16,17]. Suppression of fibrinolysis by 98% requires a TXA plasma level to be 100 μg/ml, which can be generated with a corresponding TXA regimen consisting of a loading dose of 30 mg/kg and a maintenance infusion of 10 mg/kg/hr [9,16]. To increase intrinsic generation of thrombin, a TXA plasma level of 126 μg/ml or higher may need to be sustained, which would require a TXA regimen consisting of a loading dose of 50 mg/kg with a maintenance infusion higher than 10 mg/kg/hr [9,12,13]. These effects of increasing TXA plasma concentrations on hemostasis are summarized in Figure 4.

An important concern is safety when employing of high doses of TXA. Although a high dose regimen has not been shown to increase the risk of thrombosis, very high dose TXA (100mg/kg) has been shown to increase the risk of seizures in adult cardiac surgical patients [18]. Seizures can occur when the TXA plasma levels reach 314 μg/ml, due to the inhibition of glycine receptors [19]. There were no reports of untoward side effects of high dose TXA in our study population.

Additional patient blood management strategies to mitigate blood loss were utilized, such as the use of topical hemostatic agents which is supported by evidence [23]. Cell salvage can decrease the rate of allogeneic blood transfusion from 55% to 6% in spine surgery and was also used for all surgeries in the study period [20]. Additionally, the volume of cell salvage returned to patients can be used to estimate blood loss [21]. Moderate controlled hypotension (MAP below 65) has been shown to decrease blood loss in AIS cases by 33% [22]. Our protocol for both groups included a MAP goal of 60–65 from incision until initiation of screw testing, at which time we began to increase the MAP to baseline or higher, in order to optimize spinal cord perfusion, in anticipation of rod placement and curve correction.

Some cases utilized surgical hemostasis devices. We began using a bone scalpel in 2018 and a bipolar sealer in 2019 to further reduce blood loss. The bone scalpel was used in 31 out of 126 patients in the high dose TXA group and 5 of our low dose TXA patients. The use of the ultrasonic bone scalpel has been shown to decrease blood loss by sealing the cut bone through heat emitted from the rapid ultrasonic blade [24]. The use of the bone scalpel in a larger number of our high dose TXA patients could have also contributed to our decreased blood loss and need for transfusion seen in that group. Bipolar sealers use radiofrequency energy along with saline irrigation to reduce tissue temperatures versus conventional electrocautery methods. There was no significant difference in the use of a bipolar sealer between our two study groups, but evidence does support its use in reducing blood loss during AIS surgery [25].

Placement of intraoperative halo-femoral traction in the surgical treatment of AIS has been commonly used in cases of severe scoliosis (Cobb angle >100 degrees) but can be of benefit for curves between 70 and 90 degrees as well [26]. The advantage of using halo-femoral traction include: the avoidance of a combined approach (anterior/posterior), decreased need for vertebral column resection, and reduced need for osteotomies [26,27]. Halo-femoral traction may decrease operative times by allowing for passive correction and reducing the need for extreme force to correct curves. The reduction in operating time and blood loss in the high-dose TXA group may have been at least partly due to the increased use of halo-femoral traction in this group (73% versus 32% in the low dose TXA group).

Limitations of this study include its retrospective nature, as well as the unclear contribution of bone scalpel use and halo-femoral traction on decreasing blood loss. The data was also collected over a six-year time period; therefore, surgeon experience may have contributed to faster operating times and decreased blood loss. Another potential limitation is that estimated blood loss is known to be widely variable among providers. However, our institution uses a standardized method of estimating blood loss based on the cell saver volume returned to patients in order to be as consistent as possible [21]. This is supported by the data showing that the volume of cell saver returned was higher in the low dose TXA group compared to the high dose TXA group.

In conclusion, our study provides support that the use of high dose TXA compared to low dose TXA is beneficial in reducing blood loss for patients with AIS undergoing PSF surgery. A multidisciplinary approach, with joint efforts of surgeons and anesthesiologists using this method, combined with other proven Patient Blood Management strategies, is a powerful approach in achieving the desired goal of reducing blood loss and potentially eliminating the need for transfusion. There is, however, a need for further prospective, randomized, blinded investigations to determine the optimal dosing strategy for TXA in spine surgery.

## Figures and Tables

**Table 1.** Median [25^th^, 75^th^] values for patient characteristics, surgery characteristics, anesthetic characteristics, and blood metrics and results of Wilcoxon rank-sum tests comparing low and high TXA dosing

Variable	Low-Dose TXA (N = 97)	High-Dose TXA (N = 126)	Wilcoxon P-value

**Patient Characteristics**			
Age	14 [13, 15]	14 [13, 16]	0.097
Weight (kg)	57.2 [48.8, 67.4]	55.6 [49, 64.6]	0.489
Height (cm)	161.5 [156.8, 166.6]	161.5 [156.6, 168]	0.797
Body Mass Index (BMI)	21.9 [18.6, 25]	21 [18.9, 24]	0.402
Largest Curve (degrees)	59.1 [51.4, 65.9]	60 [52.7, 69.5]	0.202
Bending Largest Curve (degrees)	33.9 [25.3, 45.3]	35.6 [27.4, 43.2]	0.558
% Curve Flexibility	39 [23.2, 55.1]	40.9 [30.5, 50.6]	0.587
**Surgical Characteristics**			
Number of Ponte Osteotomies	3 [0, 5]	4 [0, 5]	0.417
Number of Screws Placed	20 [18, 24]	20 [18, 22.8]	0.465
Number of Levels Fused	10 [9, 11]	10 [9, 11]	0.148
Post-Op Major Curve (degrees)	14.6 [9.4, 19.6]	16.8 [12.4, 24.1]	0.007
% Curve Correction	74.3 [66.5, 82.5]	70.6 [61.7, 78.9]	0.035
Implant Density	1.9 [1.7, 2]	1.9 [1.8, 2]	0.454
Operative Time (minutes)	357 [296, 399]	299 [241, 357.7]	< 0.001
**Anesthetic Characteristics**			
TXA Bolus Dose (mg/kg)	20 [18.8, 20.1]	50 [49.3, 50]	< 0.001
Controlled Hypotension MAP (mmHg)	63.2 [60, 68]	62.7 [60, 66]	0.272
Albumin Administered (grams)	37.5 [25, 50]	25 [12.5, 25]	< 0.001
Crystalloid Administered (cc/kg)	66.3 [55.5, 89.5]	58.5 [43.4, 76.2]	0.001
**Blood Metrics**			
NBL ((EBL/levels fused)/weight in kg)	2.8 [1.8, 4.4]	1.8 [1.2, 2.7]	< 0.001
EBL (cc)	1600 [1000, 3000]	1000 [600, 1500]	< 0.001
% Est Blood Volume Lost	36.3 [25.9, 71.4]	25.7 [15.7, 36.5]	< 0.001
EBL per Level Fused (cc)	160 [109.1, 272.7]	104.6 [70.4, 150]	< 0.001
EBL per Screw Placed (cc)	83.3 [55, 136.9]	50 [33.3, 75]	< 0.001
Cell Saver Returned (cc)	500 [375, 1000]	375 [250, 625]	< 0.001
Preoperative Hb (g/dl)	13.4 [12.5, 14.4]	13.5 [12.7, 14.2]	0.756
Hb at Closure (g/dl)	11.4 [10.6, 12.1]	10.7 [10.1, 11.6]	< 0.001
Lowest Intra-Op Hb (g/dl)	10.3 [9.7, 10.9]	10.4 [9.6, 11.2]	0.522
Post-Op Day 1 Hb (g/dl)	10.6 [9.8, 11.6]	9.8 [9.1, 10.7]	< 0.001
Total Drain Output (cc)	392.5 [162.5,847.8]	590 [202.3, 924.5]	0.27
Drain Duration (days)	4 [3,5]	4 [3,5]	0.49

**Table 2.** Proportions (n) for categorical patient and surgery characteristics and results of tests of association with low and high TXA dosing. P-values based on chi-square test with 1 degree of freedom unless indicated otherwise.

Variable	Low-dose TXA Prop (N=97)	High-dose TXA Prop (N=126)	P-value

Sex (% Female)	82.5 (n=80)	84.1 (n=106)	0.883
Ponte Osteotomies (% Yes)	56.7 (n=55)	57.1 (n=72)	1.0
Halo-Femoral Traction Used (% Yes)	32 (n=31)	73 (n=92)	< 0.001
Intra op PRBC Transfusion (% Yes)	67 (n=65)	19 (n=24)	< 0.001
Post op PRBC Transfusion (% Yes)	1 (n=1)	0.8 (n=1)	1.0^a^
Drain use (%Yes)	94.8 (n=92)	77 (n=97)	<0.001
Bipolar Sealer Used (% Yes)	2.1 (n=2)	9.5 (n=12)	0.026^a^
BoneScalpel Used (% Yes)	5.2 (n=5)	24.6 (n=31)	< 0.001^a^

a Fisher’s exact test
